# Supplementary material for: Formalized classification of ephemeral wetland vegetation (Isoëto-Nanojuncetea class) in Poland (Central Europe)
Source: PeerJ. 2021 Aug 10;9:e11703. doi: 10.7717/peerj.11703 (PMC8362674; doi:10.7717/peerj.11703)
Supplement: Supplemental Information 4 [file peerj-09-11703-s004.docx]

Supplementary material

Zygmunt Kącki, Andrzej Łysko, Zygmunt Dajdok, Piotr Kobierski, Rafał Krawczyk, Arkadiusz Nowak, Stanisław Rosadziński & Agnieszka Anna Popiela

Formalized classification of ephemeral wetland vegetation (Isoëto-Nanojuncetea class) in Poland (Central Europe)

**Table S2.** Merged taxa.

Taxonomic treatment in original data Taxon used in vegetation analyses.

*Anthoceros punctatus s. l.*

*Anthoceros punctatus var. crispulus*

*Anthoceros punctatus var. punctatus*

*Anthoceros punctatus s. l.*

*Arenaria serpyllifolia agg.*

*Arenaria serpyllifolia*

*Arenaria serpyllifolia agg.*

*Arenaria leptoclados*

*Arenaria serpyllifolia subsp. leptoclados*

*Arenaria serpyllifolia subsp. serpyllifolia*

*Arenaria serpyllifolia var. viscida*

*Artemisia campestris s. l.*

*Artemisia campestris*

*Artemisia campestris subsp. campestris*

*Artemisia campestris subsp. inodora*

*Artemisia campestris subsp. sericea*

*Artemisia campestris var. sericea*

*Barbula convoluta agg. -*

*Barbula convoluta*

*Streblotrichum convolutum*

*Barbula convoluta var. convoluta*

*Bolboschoenus maritimus agg.*

*Bolboschoenus maritimus*

*Bolboschoenus maritimus agg.*

*Bolboschoenus maritimus subsp. maritimus*

*Scirpus maritimus subsp. maritimus*

*Bolboschoenus laticarpus*

*Bolboschoenus planiculmis*

*Bolboschoenus sp.*

*Bolboschoenus yagara*

*Callitriche palustris s. l.* (*C. brutia subsp. hamulata* is kept separately)

*Callitriche palustris*

*Callitriche cophocarpa*

*Callitriche species*

*Callitriche stagnalis*

*Callitriche verna*

*Callitriche polymorpha*

*Carex contigua agg.*

*Carex muricata*

*Carex spicata*

*Carex muricata subsp. lamprocarpa*

*Carex muricata subsp. muricata*

*Carex pairae*

*Carex contigua*

*Carex contigua agg.*

*Carex pairaei*

*Carex flava agg.*

*Carex flava agg.*

*Carex demissa*

*Carex oederi*

*Carex lepidocarpa*

*Carex serotina*

*Carex oederi subsp. pulchella*

*Carex serotina subsp. pulchella*

*Carex serotina subsp. serotina*

*Carex viridula*

*Carex flava subsp. flava*

*Carex lepidocarpa subsp. lepidocarpa*

*Carex viridula subsp. viridula*

*Centaurea jacea agg.*

*Centaurea jacea subsp. angustifolia*

*Centaurea pannonica*

*Centaurea nigra subsp. nigra*

*Centaurea jacea agg.*

*Centaurea jacea x oxylepis*

*Centaurea jacea subsp. jacea*

*Chenopodium album agg.*

*Chenopodium album agg.*

*Chenopodium album*

*Chenopodium album subsp. viride*

*Chenopodium opulifolium*

*Chenopodium strictum*

*Chenopodium album subsp. strictum*

*Chenopodium album subsp. striatum*

*Chenopodium album subsp. microphyllum*

*Chenopodium striatiforme*

*Chenopodium strictum subsp. striatiforme*

*Chenopodium suecicum*

*Chenopodium album subsp. album*

*Chenopodium album var. lanceolatiforme*

*Eleocharis palustris agg.*

*Heleocharis palustris*

*Eleocharis palustris subsp. palustris*

*Eleocharis palustris subsp. vulgaris*

*Eleocharis palustris subsp. waltersii*

*Eleocharis mamillata*

*Eleocharis mamillata subsp. mamillata*

*Eleocharis uniglumis*

*Festuca rubra s. l.*

*Festuca rubra subsp. rubra*

*Festuca nigrescens*

*Festuca nigrescens subsp. microphylla*

*Festuca nigrescens subsp. nigrescens*

*Festuca commutata*

*Festuca rubra subsp. commmutata*

*Festuca rubra agg.*

*Festuca rubra subsp. juncea*

*Festuca arenaria*

*Festuca rubra subsp. arenaria*

*Festuca rubra var. arenaria*

*Galeopsis tetrahit s. l.*

*Galeopsis tetrahit*

*Galeopsis bifida*

*Galium palustre agg.*

*Galium palustre agg.*

*Galium palustre*

*Galium palustre s. l.*

*Galium elongatum*

*Galium palustre subsp. caespitosum*

*Galium palustre subsp. elongatum*

*Leucanthemum vulgare s. l.*

*Leucanthemum vulgare*

*Leucanthemum ircutianum*

*Leucanthemum ircutianum subsp. ircutianum*

*Leucanthemum adustum*

*Leucanthemum adustum subsp. adustum*

*Molinia caerulea s. l.*

*Molinia caerulea*

*Molinia caerulea agg.*

*Molinia caerulea subsp. caerulea*

*Molinia litoralis*

*Molinia arundinacea*

*Molinia caerulea subsp. arundinacea*

*Myosotis palustris s. l.*

*Myosotis palustris agg.*

*Myosotis scorpioides*

*Myosotis palustris subsp. palustris*

*Myosotis caespitosa*

*Myosotis laxa ssp. caespitosa*

*Plantago major s. l.* (*P. major subsp. intermedia* are listed separately)

*Plantago major*

*Plantago major subsp. major*

*Plantago major var. microstachya*

*Plantago major subsp. winteri*

*Plantago winteri*

*Persicaria lapathifolia s. l.*

*Polygonum nodosum*

*Polygonum brittingeri*

*Polygonum lapathifolium*

*Polygonum lapathifolium subsp. lapathifolium*

*Polygonum lapathifolium subsp. brittingeri*

*Polygonum pallidum*

*Polygonum tomentosum*

*Polygonum tomentosum var. incanum*

*Polygonum lapathifolium subsp. incanum*

*Persicaria lapathifolia subsp. pallida*

*Polygonum lapathifolium subsp. pallidum*

*Polygonum aviculare s. l.*

*Polygonum heterophyllum*

*Polygonum neglectum*

*Polygonum monspeliense*

*Polygonum aequale*

*Polygonum aviculare s.l.*

*Polygonum arenastrum*

*Polygonum calcatum*

*Polygonum aviculare subsp. aviculare*

*Polygonum arenastrum subsp. calcatum*

*Polygonum arenastrum subsp. arenastrum*

*Rubus fruticosus agg.*

*Rubus xcorylifolius*

*Rubus wimmerianus*

*Rubus villicaulis*

*Rubus thyrsanthus*

*Rubus tabanimontanus*

*Rubus sylvaticus*

*Rubus sulcatus*

*Rubus suberectus*

*Rubus sprengelii*

*Rubus species*

*Rubus silvaticus*

*Rubus silesiacus*

*Rubus siemianicensis*

*Rubus serpens*

*Rubus ser. Glandulosi*

*Rubus ser. Discolores*

*Rubus sect. Rubus*

*Rubus sect. Glandulosi*

*Rubus sect. Corylifolii*

*Rubus scissus*

*Rubus schleicheri*

*Rubus sanguineus*

*Rubus sanctus*

*Rubus salisburgensis*

*Rubus rudis*

*Rubus rhombifolius*

*Rubus radula*

*Rubus pyramidalis*

*Rubus pubescens*

*Rubus plicatus*

*Rubus pericrispatus*

*Rubus pedemontanus*

*Rubus parthenocissus*

*Rubus pallidus*

*Rubus orthostachys*

*Rubus opacus*

*Rubus oboranus*

*Rubus nessensis subsp. nessensis*

*Rubus nessensis*

*Rubus nemorosus*

*Rubus nemoralis*

*Rubus montanus*

*Rubus mollis*

*Rubus micans*

*Rubus macrophyllus*

*Rubus lentiginosus*

*Rubus kuleszae*

*Rubus koehleri*

*Rubus kletensis*

*Rubus josefianus*

*Rubus infestus*

*Rubus histrix*

*Rubus hirtus*

*Rubus hercynicus*

*Rubus henrici-egonis*

*Rubus hadracanthos*

*Rubus guttiferus*

*Rubus guentheri*

*Rubus grossus*

*Rubus graecensis*

*Rubus gracilis*

*Rubus grabowski*

*Rubus gothicus*

*Rubus glivicensis*

*Rubus glaucellus*

*Rubus glandulosus*

*Rubus fruticosus agg.*

*Rubus fruticosus*

*Rubus franconicus*

*Rubus fissus*

*Rubus fasciculatus*

*Rubus fabrimontanus*

*Rubus divaricatus*

*Rubus divaricatus*

*Rubus crispomarginatus*

*Rubus crassus*

*Rubus cordifolius*

*Rubus constrictus*

*Rubus clusii*

*Rubus chaerophyllus*

*Rubus canescens*

*Rubus candicans*

*Rubus brdensis*

*Rubus bifrons*

*Rubus bellardii*

*Rubus bavaricus*

*Rubus balticus*

*Rubus armeniacus*

*Rubus apricus*

*Rubus angustipaniculatus*

*Rubus acanthodes*

*Sparganium erectum agg.*

*Sparganium erectum*

*Sparganium erectum agg.*

*Sparganium ramosum*

*Sparganium neglectum*

*Sparganium erectum subsp. neglectum*

*Sparganium ramosum subsp. neglectum*

*Sparganium erectum subsp. erectum*

*Stellaria media s. l.*

*Stellaria media*

*Stellaria pallida*

*Stellaria media agg.*

*Stellaria neglecta*

*Tripleurospermum inodorum agg.*

*Tripleurospermum inodorum*

*Tripleurospermum maritimum*

*Matricaria maritima subsp. inodora*

*Matricaria maritima subsp. maritima*

*Utricularia vulgaris s. l.*

*Utricularia vulgaris*

*Utricularia australis*

*Utricularia neglecta*

*Vicia sativa s. l.*

*Vicia sativa*

*Vicia sativa subsp. sativa*

*Vicia angustifolia*

*Vicia sativa subsp. nigra*

*Vicia angustifolia subsp. segetalis*

*Viola tricolor agg.*

*Viola tricolor agg.*

*Viola tricolor*

*Viola tricolor subsp. tricolor*

*Viola tricolor subsp. ammotropha*

*Viola tricolor subsp. curtisii*

*Viola tricolor subsp. maritima*
